# Mental health in elite sports. A continuum of strategies that athletic trainers can use to support competitive athletes with psychological issues: a scoping review

**DOI:** 10.3389/fpsyg.2025.1619802

**Published:** 2025-08-11

**Authors:** Geneviève Jean-Bindley, Ana Sauriol-Gauthier, Laurie-Ann Corbin-Berrigan, Stéphanie Girard

**Affiliations:** ^1^Department of Human Kinetics, University of Québec at Trois-Rivières, Trois-Rivières, QC, Canada; ^2^University of Québec at Trois-Rivières, Trois-Rivières, QC, Canada; ^3^Groupe de Recherche sur les Affectations Neuromusculosquelettiques (GRAN), University of Québec at Trois-Rivières, Trois-Rivières, QC, Canada

**Keywords:** athletic trainers, mental health, sports, athletes, psychological support, psychological distress

## Abstract

**Introduction:**

Athletes are at high risk of experiencing psychological distress. It is unclear if professionals in charge of competitive athletes’ physical and psychological wellbeing are adequately trained to identify and manage psychological distress, despite many athletic trainers (ATs) reporting having held a position where it was necessary to counsel student-athletes on personal issues. However, ATs have also reported having received insufficient training to support athletes in the face of psychological distress.

**Objective:**

The present scoping review aims to identify, in scientific literature, how ATs can improve the psychological support provided to competitive athletes.

**Methods:**

This scoping review was carried out using a six-step process in accordance with the PRISMA-ScR guideline. The initial search, based on specific keywords related to ATs, athletes, strategies and mental health, identified a total of 1,204 articles. The research was conducted across 8 databases (APA PsycINFO, SPORTDiscuss, Google Scholar, Social Science Research Network-SSRN and others). The initial search, based on specific keywords related to ATs, athletes, strategies and mental health, identified a total of 1,204 articles. After removing duplicates, abstracts and titles were screened in accordance with the inclusion and exclusion criteria. Included articles were published between 2010 and 2024, written in English, on athletes aged 8–25 years, from Western cultures, participating in sport within a sport organization and presenting strategies that could assist ATs in the management of athletes with mental difficulties. The search yielded 22 articles that were relevant to the study.

**Results:**

Results of retained articles were separated into four themes: (1) training; (2) prevention; (3) intervention strategies; and (4) referral. Prevention involves implementing a set of measures aimed at avoiding or reducing the number and severity of psychological distress. Training helps fill gaps in the understanding and application of psychological interventions in sport. Intervention strategies allow professionals to intervene based on meeting the fundamental psychological needs of athletes. Finally, as soon as signs of psychological distress are identified, it is important to refer the athlete to a mental health professional in order to provide adequate support to athletes.

**Discussion and conclusion:**

Based on the results, two themes are most often reported, such as prevention and intervention strategies. Future research should explore the long-term impacts of these interventions implemented by ATs on athletes’ mental wellbeing.

## 1 Introduction

Psychological distress manifests itself primarily through internalized symptoms, such as depression and anxiety (Bélanger et al., 2024; Finn, 2016). Although it is not classified as a psychological mental health disorder, distress still indicates a sense of suffering (Union Étudiante Du Québec, 2019). Concerns about mental health have grown in the post-COVID-19 era, particularly regarding the increasing prevalence of reported psychological distress among youth in various contexts, including sports (Yard et al., 2021). This increasing prevalence highlights mental health challenges as a growing area of concern in the field of sport (Rice et al., 2016). Indeed, athletes face immense pressure to succeed, among other stressors and challenges, in the setting of competitive sport, and this burden only intensifies as they progress to higher competition levels (Arnold et al., 2016). Increased internal and external expectations from coaches, parents, teammates can lead to heightened stress, anxiety, and mental exhaustion in athletes (Burke et al., 2023). In addition, this pressure is amplified by external factors such as media scrutiny, scholarship or sponsorship requests, and the need to balance academic or personal life with training. Academic struggles, poor nutrition, fatigue, lack of role models, and financial insecurity are just a few of the factors that can impact a student-athlete’s mental health (Cosh and Tully, 2015; Book et al., 2022). This constant pursuit may lead to psychological distress, affecting both mental wellbeing and overall sport performance (Arnold et al., 2016; Butler-Coyne et al., 2019; Burke et al., 2023) and manifesting as a wide range of symptoms including, but not limited to depression, anxiety, sleeping disturbances, adverse alcohol and nutrition behavior (Gouttebarge et al., 2015). Mental health challenges in sport environment are a common occurrence for athletes, and one might argue that the demands of our performance-driven society, the rising pressure of training and performance as well as the perception toward athletes as being strong and independent individuals may contribute in part to their psychological distress (Miller, 2018; Gwyther et al., 2024).

Psychological distress experienced by athletes can radiate onto people in their close environment. Indeed, it is suggested that professionals working alongside athletes, such as coaches and medical personnel, are exposed to a high proportion of athletes who are experiencing psychological distress (Ayala et al., 2024). In addition, research highlights the role of sports professionals regarding athlete’s mental perceptions of challenges (Ayala et al., 2024). For instance, Barcza-Renner et al. (2016) suggested that the risk of burnout in athletes correlated with controlling coaching behaviors experienced by athletes. Nevertheless, it is often found that athletes do not have access to adequate psychological support (Miller, 2018). In fact, despite a plethora of professionals in charge of elite athletes’ physical and psychological wellbeing, such as coaches, physical therapists, and athletic trainers (ATs), not all professionals are trained or qualified in this area of expertise. The present work will focus on the role of ATs with regards to athletes’ wellbeing as they are particularly well-positioned to identify and address struggles in athletes. By the NATA (2021, para. 2) definition:

Athletic trainers (ATs) are highly qualified, multi-skilled health care professionals who render service or treatment, under the direction of or in collaboration with a physician, in accordance with their education, training and the state's statutes, rules and regulations. As a part of the health care team, services provided by athletic trainers include primary care, injury and illness prevention, wellness promotion and education, emergent care, examination and clinical diagnosis, therapeutic intervention and rehabilitation of injuries and medical conditions.

In this respect, ATs have the potential to develop a close relationship with athletes both in the day-to-day aspects of training and in the demands of competition, making them key individuals in identifying struggling athletes (Biviano, 2010); potentially allowing for proper care trajectory.

Even though ATs are highly trained healthcare professionals, their primary area of expertise resides in the physical system rather than in the psychological domain. In the context of competitive sports, it is put forward that sport professionals in proximity to athletes, such as ATs, may play a bigger role on psychological health than expected (Ayala et al., 2024). Due to this proximity, it is reasonable to believe that ATs are also key individuals for athletes to confide in. However, a number of ATs claims that they are not confident in their capacity to provide mental health care and that they have not been adequately trained to handle psychological discomfort (Cormier and Zizzi, 2015; Anderson et al., 2023). Furthermore, there appears to be a wider gap in knowledge and ease in the handling of psychological discomfort that is not injury-related in the practice of ATs (Cormier and Zizzi, 2015). This lack of comfort in identifying and addressing distress in athletes may come from ATs’ training cursus, leading as suggested by ATs reporting having received insufficient training to support athletes when facing psychological distress (Cormier and Zizzi, 2015). Despite reporting insufficient specific training on the topic of distress, it is suggested that ATs might have basic level training in the matter, from having learned strategies that could be applied to distress. As such, some psychological support strategies for ATs related to physical impairments, like facilitating the athlete’s understanding of the injury, setting rehabilitation goals, encouraging effective communication, and using active listening, are known to be effective (Cormier and Zizzi, 2015). While ATs are not, by definition, licensed mental health providers, they do, very often, find themselves in first responder positions to signs of psychological distress. Understanding this within an interdisciplinary context is essential for effective support. However, much remains to be done when it comes to management of non-injury-related psychological distress.

To face psychological distress in athletes, some tools have been made available to ATs and other health care professional to identify mental health issues, such as self-reported questionnaires and other screening tools (Cormier and Zizzi, 2015). However, the propensity to use these tools remains unknown. It is however established that the confidence and ease on the topic of psychological distress is closely related to the likelihood of adequate referral in health care practitioners. As such, there is a significant positive association between the frequency of athlete referrals to sport psychologists and ATs feeling more comfortable and able to discuss non-injury related psychological issues with athletes (Biviano, 2010; Cormier and Zizzi, 2015). This could indicate that ATs who do initiate discussion about psychological problems are more likely to correctly refer an athlete to a sport psychologist, for example (Biviano, 2010; Anderson et al., 2023). In addition, research indicates that ATs are inclined to refer athletes to specialists, such as sports psychologists, when they feel comfortable discussing mental health (Biviano, 2010; Anderson et al., 2023). While ATs’ comfort in managing mental health concerns has improved due to training programs like Mental Health First Aid (MHFA), additional education is essential to enhance their role in providing psychological support (Anderson et al., 2023). According to Cormier and Zizzi (2015, p. 1271), “*97.3% of respondents believed that facilitating psychological referrals was a major responsibility of all ATs when athletes exhibited personal distress.*” However, when it comes to addressing moderate-level symptoms, ATs tend to struggle with determining the appropriate action to take (Biviano, 2010; Cormier and Zizzi, 2015). It is therefore important for ATs to develop the knowledge, skills and resources they need to provide psychological support and guidance to athletes in need. Despite the increasing recognition of mental health concerns in athletes, there is limited research on evidence-based interventions that ATs can effectively integrate into their practice. Considering the above, the present scoping review aims to explore how ATs can improve the psychological support they provide to competitive athletes.

## 2 Methods

A scoping review was conducted to provide a comprehensive review of the existing scientific literature, allowing for examining a diverse and emerging body of evidence. This scoping review was conducted in accordance with PRISMA extension for scoping review (PRISMA-ScR) (Page et al., 2021) following a 5-steps (and 6th optional, not presented in this review) process outlined by Mak and Thomas (2022).

### 2.1 Stage 1: identifying the research question

The present scoping aimed to review how ATs can improve the psychological support provided to competitive athletes. The research question associated with this aim was: How can ATs improve the psychological support provided to competitive athletes?

### 2.2 Stage 2: identifying relevant studies

The searches were conducted in a series of incremental periods between October and September 2024, with search terms generated in consultation with research experts and an expert librarian specializing in the field of physical activity and rehabilitation. These terms were designed to relate to the population of interest (e.g., “athletes” OR “teens players” OR “young athletes” OR players OR sportsmen OR sportswomen), as well as the outcomes measures (e.g., approaches OR strategies OR assistance OR intervention OR action OR treatment OR assistance OR mediation OR “therapeutic intervention” OR “emotional support” OR “supportive care” OR “patient support” or “psychological support” OR “social support”), the psychological topics (e.g., “mental disorder*” OR “psychological distress” OR “Psychological issues” OR “mental challenge*” OR “psychological problem*” OR “psychological concern*”) and the professional personnel (e.g., “Athletic trainer*” OR coach* OR trainer* OR kinesiologist OR “strength coach” OR physiotherapist OR “mental preparator” OR “sport psychologist” OR “mental coach” OR “Sports therapist”).

## 3 Results

### 3.1 Stage 3: selecting studies to be included in the review

To be included in the scoping review, the articles had to meet eligibility criteria depicted in Table 1.

**Table 1:** Justifications of the eligibility criteria.

Themes	Inclusion criteria	Exclusion criteria	Justification
Population	Athletes aged between at least 8–25 years old in Western cultural contexts.	Athletes from other cultural contexts and younger than 8 years old or older than 25 years old.	The athlete population is relatively young: 79.1% of the sports population is between 4 and 29 years old, and <19% are aged between 5 and 9 years old (Eime et al., 2016). The results focus on settings where healthcare systems, cultural norms, and access to resources do not differ significantly, ensuring relevance to the target audience.
	Athletes that are part of a sport organization.	Athletes who are not part of a sports organization.	ATs rarely intervene with non-athletes in their practice. Young athletes who are not affiliated with a sports organization typically have limited access to AT.
Intervention outcomes	Strategies that can be of assistance to ATs in the management of athletes with mental challenges.	Interventions focusing on diagnosis, screening, or screening tools.	Identifying intervention strategies that directly address psychological management. Including diagnosis and screening tools could also overcomplicate the review. These topics are also already covered in the literature.
	Interventions centered on psychological challenges in sports.	Interventions were aimed at performance enhancement rather than psychological health.	Athletic therapists are primarily trained to address health-related issues (e.g., injuries, prevention and rehabilitation). Existing guidelines already cover performance-enhancing interventions.
		Studies related to health issues (e.g., concussions) addressing topics such as gender, cultural or religious identities, or to specific context (e.g., COVID-19).	It was important to have results that could be generalized to typical athletic therapy practices. Studies that deal with niche topics or in unusual contexts may not apply to the standard practice.
Context			
Language	Articles published in English.	Articles published in languages other than English.	English is one of the most common languages used in research and publishing journals (Balan, 2021). Ensuring accessibility and consistency in the review process.
Study outcomes	Qualified professionals using applicable strategies in the scope of practice of ATs.	No mention of strategies applicable for ATs practices or the involvement of a qualified professional.	Outcomes must align with the scope of practice of ATs or be achievable by them. The goal of this review is to offer value for practitioners seeking to improve ATs practices and to connect practices that are related to sports therapy to make relevant connections with similar sports health practices, studies and stakeholders.
Timeframe	Articles published between 2010 and 2024.	The publications were before 2010 and after 2024.	Health science research is fast evolving. Using outdated research could lead to recommendations that no longer align with best practices nor with sport therapy context, which is a recent field of interest.

ATs, Athletic trainers.

**Figure 1:**
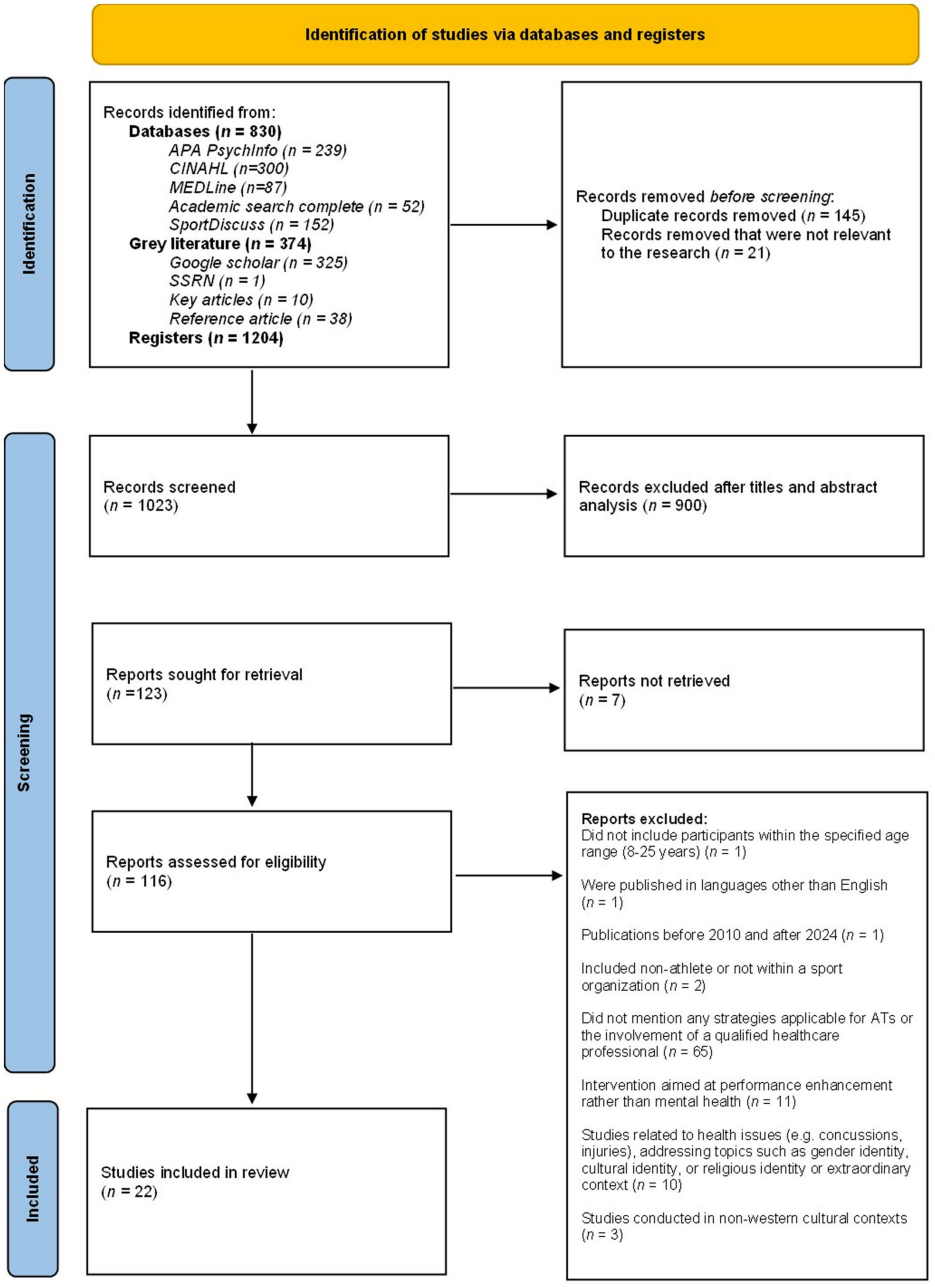
PRISMA 2020 flow diagram for systematic reviews and scoping reviews. ATs = Athletic trainers.

The searches yielded a total of 1,204 articles to be screened for eligibility. After removing duplicates, 1,059 articles remained. In addition, 21 articles were removed, because they did not fit the area of interest of the research, resulting in 1038 articles to be screened. Abstracts and titles were screened first, leaving a total of 123 potential articles. Seven articles were not retrieved, because they were not available for consultation. The remaining articles (*n* = 116) were then screened by retaining only those that met inclusion criteria by two independent reviewers (ASG and GJB), as displayed in the PRISMA Flow chart (Figure 1). Reviewers first screened the titles, then the abstracts, and next confirmed that the results of the articles were in line with the proposed review and that they put forward strategies. They then discussed any disagreements and, if they could not reach a consensus, a third author and experienced researcher (SG), acted as a moderator and made the final decision. This process led to a total of 30 articles to be included in the review and read in full. Upon reading the articles in full, those that did not meet the inclusion criteria were removed (*n* = 8), leaving a total of 22 articles being selected for the review.

### 3.2 Stage 4: charting the data

Analysis grids for data extraction were developed by the research team and were used to provide a systematic method for reading the 22 articles in depth. Demographic elements of the articles were extracted by one reviewer (GJB), including sample age (if available), type of participants, study design, cultural context, sports (if available) and types of intervention. Results of each article were analyzed qualitatively and were classified into four themes: (1) training; (2) prevention; (3) intervention strategies; and (4) referral. Results of the third theme, Intervention strategies, were separated into four subthemes: (1) need-supportive climate; (2) communication; (3) involve the entourage; and (4) professional quality. Definitions of each theme were established to help with classification. In the context of this study, *Training* referred to formal courses or activities that are designed to build specific competencies, knowledge, or skills that are relevant to the topic of the research. *Prevention* referred to an indication to initiate a service, an action and a complementary approach that could help prevent a psychological crisis in athletes. As for *Intervention strategies*, findings were extracted regarding how ATs could intervene during a direct interaction with athletes manifesting psychological distress. Finally, *Referral* implied that, in certain cases, it was suggested that other professionals be involved in the process for the benefit of athletes.

Table 2 presents authors, country, psychological issues addressed, study design and the main results according to each theme. Quality assessment of included studies, not required in a scoping review, was not performed, due to the wide range and heterogeneity of included study designs.

**Table 2:** Study characteristics and key results.

Articles	Country	Participants	Issues	Design	Results according to each theme: (1) training; (2) prevention; (3) intervention strategies; (4) referral
1. Bengtsson et al. (2024)	USA, Australia, Spain	No direct participants.	Motivation, anxiety, self-esteem, enjoyment, and social cohesion.	Systematic review and meta-analysis.	(1) Interpersonal coach and parent development programs CDP and PDP highlight the importance of coaches’ interpersonal skills for shaping the youth sports environment based on four different approaches (presented below). (2) and (1) Coach Effectiveness Training (CET) Mastery Approach to Coaching Need-Supportive Coaching Autonomy-Supportive Parenting
2. Brenner and Watson (2024)	USA	Child and adolescent athletes.	Burnout.	Clinical report.	(2) Recommendations for prevention: Promote the conduction of preparticipation examinations within the athlete’s medical environment to afford a more comprehensive approach to young athlete care that can incorporate guidance regarding overuse injuries, overtraining, and burnout. Encourage athletic autonomy and intrinsic motivation: measure success on participation and effort. Foster positive experiences with parents, coaches, and peers, all of which can help prevent burnout. Promote skill development and being well-rounded in physical activities while avoiding overtraining and overscheduling. Encourage the athlete, parent, and coach to modify the causative factors and involve mental health professionals, if needed. Encourage mindfulness tools. Focus on wellness and teaching athletes to listen to their bodies.
3. Braun and Tamminen (2019)	Canada	Five male coaches and 10 athletes (6 girls) in individual varsity sports (fencing, swimming, track and field, Nordic skiing and squash).	Emotion regulation.	Longitudinal multiple case study approach.	(3) Regulate athletes’ emotions: Attentional deployment: Attempts to shift athletes’ attention toward or away from a situation. Goal setting: set process goals with athletes Cognitive change: use reappraisal strategies to try and modify an athlete’s interpretation of a situation in competition and practice. Response modulation: use of positive reinforcement among athletes who were expressing negative emotions. Listening to when athletes vent their emotions. Closeness and commitment. Communicating with each other is crucial for giving athletes tools they need.
4. Campbell et al. (2022)	No specific country Authors are affiliated in USA and Ireland	No direct participants.	Motivation, engagement, burnout, dropout, Emotional wellbeing, coach-athlete relationships.	Conceptual and practical application article.	(2) and (3) Use motivational strategies to create a motivational climate and meet the basic needs of athletes such as Self-determination theory (SDT): Need-thwarting coaching styles: behaviors that hinder or frustrate athletes’ need for autonomy, competence and relatedness. Need-supportive coaching styles: behaviors that promote athletes’ basic need for autonomy, competence and relatedness. Promote autonomy: Provide choice. Use noncontrolling, informational language. Orient to intrinsic values and goals. Provide a meaningful rationale. Promote competence: Provide structure. Set optimal challenges. Offer constructive, clear and relevant feedback. Promote self-monitoring. Foster relatedness: Taking the athletes’ perspective. Demonstrate an interest in athletes as people. Prompt athletes to ask questions or contribute to discussions. Use empathetic listening. Provide opportunities for ongoing support.
5. Curran et al. (2016)	United Kingdom	252 soccer athletes (M_age_ = 12.98; SD = 1.84; girls = 67)	Motivation, engagement, psychological needs satisfaction and frustration.	Three-wave longitudinal design.	(3) Use motivational strategies to create a motivational climate meeting the basic psychological needs of athletes (SDT).
6. Gwyther et al. (2024)	No specific country Authors are affiliated in Australia, Canada and USA	No direct participants.	Disordered eating, anxiety, depression, substance use, burnout, wellbeing and stress management.	Scoping review.	(3) Have safe and open discussions and develop positive caregiver relationships.
7. Henriksen et al. (2014)	Denmark	4 sport psychology practitioners.	Not mentioned.	Qualitative interview approach.	(2) Provide young athletes with a holistic skills package that includes, but also goes beyond, mental skills and a variety of psychosocial skills, not all of which are directly related to sport: Goal setting. Coping with adversity in sports and life. Handling injuries and other challenges in the transition from junior to senior level. Prioritizing and planning daily life and balancing sport, school, recovery, and social life. Be aware of the social network as a resource. (3) Help the athletes to gradually build a professional attitude by helping them distinguish between factors within or outside their control. (3) Involve athletes’ environment like family, coaches, and sometimes relevant peers as much as possible in interventions and informing them of the purpose and focus of interventions.
8. Huynh et al. (2020)	USA	184 undergraduate students (*M*_age_ = 23.44; girls = 73.4%).	Psychological trust, emotional trust, cognition-trust.	Quantitative survey-based design.	(3) Have humility: Display a secure accepting identity, a self-view that is free from distortion, and an openness to new information.
9. Jasser et al. (2022)	USA	No direct participants.	Anxiety, depression, substance use, performance-enhancing drugs, overtraining syndrome, eating disorders, sport-related injuries and mental health.	Review and policy recommendation article.	(2) Do a mental health screening. Annual check-ups present a routine opportunity for screening, as do visits related to a specific concern.
10. Jiménez et al. (2019)	Spain	18 elite swimmers (M_age_ = 15.3; SD = 1.86: girls = 10)	Stress and cortisol response.	Experimental study.	(3) Have a democratic approach: Collaborative approach Encourage athlete participation Positive dialogue Group cohesion Foster self-confidence, resilience, and lower stress levels
11. Kipp (2014)	USA	303 gymnasts (M_age_ = 13.0; *SD* = 1.9; girls = 303)	Self-esteem, positive affect, disordered eating.	Dissertation cross-sectional design.	(3) Use motivational strategies to create a need-supportive motivational climate (SDT): Foster autonomy. Foster mastery climate (emphasizing effort, improvement and choice). Foster relatedness (feeling connected to coaches).
		A subsample: 174 gymnasts (M_age_ = 13.5; *SD* = NA; girls = 174)		Longitudinal design.	
12. Laborde et al. (2017)	Spain	2,135 sports coaches in individual and team sports (M_age_ = 31.10; SD = NA; girls = 510; boys = 1,625)	Coping strategies, stress, self-esteem, burnout	Cross-sectional observational design.	(3) Have positive personality traits: Hope. Optimism. Perseverance. Resilience.
13. Langan et al. (2015)	Ireland	87 football athletes (EG: M_age_ = 15.02; SD = 1.62; CG: M_age_ = 15.34; SD = 0.96)	Motivation and burnout.	Cluster randomized controlled trial (RCT).	(3) Focus on intervention that increases need-supportive behavior and reduces controlling behavior (SDT).
14. Lopes Dos Santos et al. (2020)	USA	No direct participants.	Sources of stress, monitoring strategies, coping mechanisms.	Narrative review.	(2) Build and managing coping strategies: Teach some basic skills like introducing athletes to basic lifestyle concepts, such as practicing deep breathing techniques, positive self-talk, and developing healthy sleep habits. Establish an open-door policy wherein the team members feel comfortable approaching staff to seek out resources for coping with challenges related to stress. Use subjective measures for stress monitoring, like RESTQ-S and POMS. Promote autoregulation in training. Encourage social support. Do regular check-ins. (4) Refer collegiate athlete to a sport psychology or other mental health consultant when the interventions aiming to improve mental health expand from basic concepts to mental training.
15. Neal et al. (2015)	USA	No direct participants.	Depression, anxiety, stress, eating disorders, substance use, suicide risk, identity challenges, academic and athletic pressures.	Consensus statement providing guidelines and recommendations.	(2) (4) Have a plan to recognize and be able to refer student-athletes with psychological concerns. Assist the student-athlete in accessing the mental health care system. (2) Monitor behaviors: Before arranging a private meeting with the student-athlete, it is important to have accurate facts, with context, relative to the behavior of concern. (3) The conversation should focus on the student-athlete as a person, not as an athlete. Listening empathetically and encouraging the student-athlete to talk about what is happening are essential. Use open-ended questions. Point out that mental health is as important as physical health.
16. Lawrence and Gunter (2023)	USA	No direct participants.	Mental health challenges.	Review and policy recommendation article.	(2) Develop a Mental Health Emergency Action Plan.
17. Sebbens et al. (2016)	Australia	166 coaches, trainers, support staff and service providers (e.g., nutritionists, physiotherapists), managers and administrators (M_age_ = 37,8; SD = 10.6; girls = 83; boys = 83)	Mental illness.	Quasi-experimental design.	(1) Mental Health in Sport (MHS) workshop is an intervention designed to improve mental health literacy and confidence in elite sport staff. Coaches and staff are trained to: Identify symptoms of mental illness. Initiate supportive conversations. Refer athletes to professional help.
18. Tamminen et al. (2012)	Canada	No direct participants.	Psychosocial challenges, disordered eating, alcohol and drug abuse.	Literature review.	(2) Create a supportive context: Question and remind athletes about effective coping strategies. Provide perspective. Share personal experiences. Dose or structure potentially stressful experiences for athletes. Initiate informal conversations about coping. Create learning opportunities, and direct instruction about coping. (2) Adopt positive behaviors: Provide social support. Use positive communication. Develop psychological and social skills. Keep sport in perspective.
19. Tamminen and Holt (2012)	Canada	34 participants: 17 athletes, 10 parents, and 7 male coaches (M_age_ = 15.6; SD = NA)	Stress.	Grounded theory methodology.	(2) Establish a “psychologically safe” environment for athletes to feel comfortable discussing stressors and allow coping: Listen and monitor athletes’ reactions. Monitor their own reactions to situation, Read athletes’ body language and responsiveness. Foster independence: balance between supporting young athletes in becoming independent in coping with stressors and protecting athletes who have difficulty coping. Question and remind athletes about effective coping strategies Provide perspective Share personal experiences Dose or structure potentially stressful experiences for athletes Initiate informal conversations about coping Create learning opportunities, and direct instruction about coping
20. Neal et al. (2013)	USA	No direct participants.	Mental health concerns among student-athletes, psychological distress, impact of injuries on mental health, suicide prevention, barriers to seeking help.	Policy and guidance document based on literature review, expert opinions, and existing policies related to mental health in college sports.	Develop a plan for the recognition and referral of student-athletes with psychological concerns: (2) Preparticipation physical examination of mental health concerns. Obtain and have readily available an institution-based plan and evaluation protocol for new and emerging mental health situations. Follow the protocol. Monitoring behaviors. (3) Approach the student-athlete with a potential psychological concern. Foster empathetic listening Encourage the student-athlete to talk about what is happening. Focus on the athlete as a person. Inform the student-athlete about the aspect of confidentiality. (4) Have a routine referral for mental health evaluation. Refer the student-athlete for psychological evaluation and care. Refer new and emerging mental health situations.
21. Wilczyńska et al. (2021)	Poland	8 coaches (football = 6; gymnastics = 2), 57 athletes (gymnastic = 23 girls; football = 34 boys) (EG: M_age_ = 9.6; SD = 1.1; CG: M_age_ = 10.3; SD = 0.9)	Motivation, anxiety, coach-athlete relationship.	Pilot controlled trial.	(1) Psychological Workshops based on the i7W model, which focuses on improving coach-athlete relationships. This model focuses on the coach-athlete relationship to promote psychosocial development and spors achievements and is based on specific principles: inspire, explain, expect, support, reward, and appreciate.
22. Zajonz et al. (2024)	Germany	57 coaches from individual sports (e.g., running, swimming, triathlon) from the recreational to international level (M_age_ = 40,93; SD = 14,24; girls = 12; boys = 45)	Emotional intelligence.	Experimental study with randomized controlled design.	(1) Online-based emotional intelligence (EI) training specifically designed to address coaches’ needs on different dimensions of coaching efficacy.

M, Mean; EG, Experimental group; CG, Control group; SD, Standard deviation; NA, Not applicable; RESTQ-S, Recovery Stress Questionnaire for Athletes; POMS, Profile of Mood States.

### 3.3 Stage 5: collating, summarizing, and reporting the results

Included articles were published between the years 2013–2024. The vast majority (63.6%) came from North American research groups. The psychological issues that were the most studied were motivation (22.7%), anxiety (22.7%) and disordered eating (22.7%). In nine articles (40.9%), no participants were directly involved. In studies including participants, one (4.5%) was an analysis of the responses of 10 parents to an intervention, one (4.5%) was a survey of four mental health professionals, one (4.5%) examined the responses of 184 undergraduate students to sport coaching styles. Finally, six articles (27.3%) investigated the responses of a total of 2,378 coaches. Next, qualitative results are presented according to each theme and subtheme.

### 3.4 Training

As seen in Table 2, five (22.7%) articles cited training on the topic of how to deal with mental health and distress as a necessary tool for learning adequate management skills. Learning psychological skills and communication adroitness, with training such as the interpersonal coach and parent development programs (CPS and PDP) and workshops to improve mental health literacy (Coach Effectiveness Training-CET, Mastery Approach to Coaching, Need-Supportive Coaching, Autonomy-Supportive Parenting and others) was found to help shaping youth environment, increase perceived task-related motivational climate (Wilczyńska et al., 2021; Bengtsson et al., 2024), athletes’ self-determination motivation (Wilczyńska et al., 2021; Bengtsson et al., 2024), team social cohesion (Bengtsson et al., 2024), professional’s depression and anxiety literacy (Sebbens et al., 2016) as well as confidence for intervention (Sebbens et al., 2016). Specifically, the workshops evaluated in the study of Wilczyńska et al. (2021, p. 587) “*aimed to increase the coaches’ ability to use the behavioral dimensions of the i7W model: inspire, support, explain, expect, reward, and appreciate*.” In a group of gymnasts, it was shown that after taking part in these workshops, even though there was a significant increase of gymnasts’ motivation, results were mitigated: there were unclear and harmful effects on anxiety state and on performance (reaction-time) of young athletes. Despite this conflicting evidence, both Bengtsson et al. (2024) and Sebbens et al. (2016) suggested that training, such as CPS, PDP and Mental Health in Sports Workshop could help professionals decrease anxiety, levels of burnout, somatic tensions, concentration disruption and worry in athletes. Finally, emotional intelligence training program helped to improve professionals’ interpersonal emotional competencies (Zajonz et al., 2024). The training mentioned by Bengtsson et al. (2024) are also used as a means of prevention.

### 3.5 Prevention

Eleven (50%) articles identified prevention strategies to support athletes before psychological issues arise. For instance, establishing procedures, standards and politics to approach athletes during mental distress, such as using a need-supportive approach (e.g., mastery and autonomy support), could help prevent situation that relate to psychological issues (Campbell et al., 2022; Bengtsson et al., 2024; Brenner and Watson, 2024). Moreover, it is suggested that using prevention strategies encourages athletes to solicit the involvement of different mental health professionals, when and if needed (Brenner and Watson, 2024), which is a good support’s practice (Neal et al., 2013; Neal et al., 2015; Lopes Dos Santos et al., 2020).

According to the retained studies, prevention strategies can be applied in different ways and provide many benefits for athletes. For example, measuring success on participation and effort and fostering positive experiences with peers encourage athletic autonomy and intrinsic motivation as well as skill development and being well-rounded in physical activities, while preventing burnout, and avoiding overtraining and overscheduling (Lopes Dos Santos et al., 2020). In addition, using mindfulness, promoting wellness and teaching athletes to listen to their bodies help them to build basic lifestyle foundations, such as breathing techniques, healthy sleep habits, and positive self-talk, that can enable them to manage stress (Lopes Dos Santos et al., 2020; Brenner and Watson, 2024).

In the same line, Henriksen et al. (2014) suggested that equipping young athletes with a holistic skill set that extends beyond traditional mental training, like incorporating essential psychosocial competencies, is beneficial in contexts pertaining both athletic and personal life. This collection of abilities emphasizes goal setting, resilience in the face of adversity, managing injuries and transitions, balancing sport with academic and social commitments, and leveraging social networks as a support system (Henriksen et al., 2014). Similarly, autoregulation can also be practiced by athletes, allowing them to modulate workload according to their own perceptions giving them the chance to modulate their workloads according to their general perceptions (Lopes Dos Santos et al., 2020). Again, this practice can be applied both in training and personal life.

Some articles recommended promoting the conduction of preparticipation examinations within the medical home, referring to medical support system around athletes, to afford a more comprehensive approach to young athlete care that can incorporate guidance regarding overuse injuries, overtraining, and burnout (Neal et al., 2013; Jasser et al., 2022; Brenner and Watson, 2024). This approach leads to more effective tools for attending mental health needs, which enhances overall outcomes for athletes (Jasser et al., 2022). To prevent athletes’ psychological distress, athletic trainers are often involved in the development of a mental health emergency action plan (Lawrence and Gunter, 2023). Indeed, when dealing with mental emergencies of student-athletes, the consensus statement entitled *Inter-association for developing a plan to recognize and refer student-athletes with psychological concerns at the collegiate level* recommend developing a plan and protocol for emergent mental health evaluations, which provide the steps to follow and support the decision making (Neal et al., 2013). To do so, the plan should include local mental health resources near the institution and make provisions for follow-up care. Furthermore, athletic trainers must also educate the athletes and staff in the event of mental health issues that would prompt early interventions with coping skills (Lawrence and Gunter, 2023).

Finally, there are some research-based strategies that professionals can use to help athletes develop healthy coping strategies and resiliency (Tamminen et al., 2012). Some examples of practical interventions include modeling through shared experience, dosing stressful interventions, guiding reflection, doing a task breakdown of difficult situations, initiating informal learning periods and doing explicit teaching of task-oriented coping mechanisms (Tamminen et al., 2012; Tamminen and Holt, 2012). In addition, it is recommended that coaches and other stakeholders adopt an open-door policy and have safe and open discussions with athletes (Neal et al., 2013; Neal et al., 2015; Lopes Dos Santos et al., 2020). In doing so, it could contribute to reducing student-athletes’ psychological burdens related to performance pressure, injuries, and disputes or disagreement with teammates or even coaches (Neal et al., 2015; Lawrence and Gunter, 2023). In a grounded theory article, Tamminen and Holt (2012) added that coaches with enhanced introspection, with regards to listening and monitoring their own reaction, help foster safe conversation. It is also suggested that reading the athletes’ body language and responsiveness incites athletes to be more receptive to feedback, and that fostering independence incites a sense of competence in athletes. In contrast, unsupportive coaches and need thwarting coaching style led to disengagement, maladaptive coping strategies (e.g., rumination, disordered eating, substance abuse, and steroid use) and frustration of athletes’ basic psychological needs (Tamminen et al., 2012; Campbell et al., 2022). Indeed, Gwyther et al. (2024) suggested that caregiver, peer, and coach relationships could be both protective and risk factors of mental distress in athletes. As such, creating a protective environment and facilitating open discussions between athletes and their social environment, including but not limited to partners and coaches, have been correlated with reduced disordered eating symptomatology in athletes (Scoffier et al., 2010; Gwyther et al., 2024). On the other hand, external pressures regarding body image and weight coming from the coaches and the peers have been correlated with greater symptom severity (Gwyther et al., 2024).

### 3.6 Intervention strategies

Ten (45.4%) studies proposed intervention strategies as a valuable solution to athletes’ manifesting psychological distress. These intervention strategies were summarized into four subthemes: (1) creating a need-supportive climate; (2) encouraging communication; (3) requesting the involvement of the entourage; and (4) highlighting the importance of developing specific professional qualities.

#### 3.6.1 Need-supportive climate

Three (13.6%) studies demonstrated that creating a need-supportive motivational climate, as stipulated by the self-determination theory (SDT; Ryan and Deci, 2017), improved coach-athlete relationship (Kipp, 2014; Langan et al., 2015; Curran et al., 2016). Curran et al. (2016) suggested that coaching motivational style (autonomy support and interpersonal control) is a predictor of athletes’ basic psychological need satisfaction. Specifically, autonomy support was positively related to athletes’ engagement, and negatively to disaffection and frustration during the sporting season. However, if coaches used interpersonal control at the beginning of the season, it was negatively associated with engagement and satisfaction in athletes (Curran et al., 2016). Similarly, SDT-based interventions have shown promising results in positive changes in coaches’ behaviors (mastery or autonomy-support), which lowered burnout in athletes (Langan et al., 2015), had a positive effect on disordered eating, psychological need satisfaction (autonomy, competence and relatedness) and athletes’ wellbeing (self-esteem) (Kipp, 2014).

#### 3.6.2 Communication

Encouraging the regulation of athletes’ emotions by means of communication generates positive reinforcement (Laborde et al., 2017; Braun and Tamminen, 2019). Supporting athletes regulate their emotions by shifting their attention, setting goals, using open-ended questions, using reappraisal strategies and positive reinforcement (e.g., hugs, high five and encouraging words), was found to calm anxiety (during and after competition), ease frustration or disappointment and lessen stigma about mental health in sports (Neal et al., 2013; Neal et al., 2015). Indeed, a professional expertise, like being able to identify and differentiate between what is within athletes’ control (e.g., effort, attitude, preparation) and what is outside of their control (e.g., opponents, other people’s judgments, weather), can also help athletes cope with adversity, be task oriented, stay motivated and handle other challenges, like injuries in sport and life (Henriksen et al., 2014). Furthermore, a positive coach-athlete relationship was also found to help athletes modulate their emotions (Laborde et al., 2017; Braun and Tamminen, 2019). Indeed, coaches using adaptive coping strategies, such as calming emotions and planning, foster an environment in which athletes may more easily manage stress. For example, in a team climate where coaches promote social support and calmness, athletes feel encouraged to seek help and to manage their emotions (Laborde et al., 2017). Alternatively, a reliance on mental withdrawal in the coaching process may foster an environment where athletes feel less supported and rather isolated in their coping (Laborde et al., 2017). Furthermore, during key moments (e.g., during workout), using positive reinforcement ensures a greater positive effect on athletes’ emotions (Braun and Tamminen, 2019).

According to many authors, communicating using empathetic listening and highlighting the aspects of confidentiality around discussions are important to have safe and open discussions and develop positive caregiver relationships (Neal et al., 2013; Neal et al., 2015; Braun and Tamminen, 2019; Gwyther et al., 2024). Finally, focusing on the athlete as a person and not just an athlete was a key factor for successful intervention (Neal et al., 2015). Counterintuitively, Braun and Tamminen (2019) also presented some strategies used by coaches such as yelling and criticism to trigger or enhance athletes’ emotions, such as frustration and anger. This strategy was used infrequently to ensure it did not harm the athletes, but it was suggested that it may have helped them to be more driven (Braun and Tamminen, 2019).

#### 3.6.3 Involve the entourage

It is strongly suggested that incorporating stakeholders such as family members, coaches, and peers into the intervention process enhances a facilitative environment. By sharing the intent, the objective and the goals, it reaffirms the athletes’ development and growth (Henriksen et al., 2014). In fact, for coaches, being able to understand and make athletes aware of their mental workload, as a role model, contributes to managing it positively (Henriksen et al., 2014). This can relate to Gwyther et al. (2024) which suggests that isolation could be detrimental for athletes. Furthermore, the relationship with the coach was a predictor of self-esteem and body image in athletes (Gwyther et al., 2024). According to Henriksen et al. (2014), not only is it important for practitioners to be present, but they must also be involved in the athlete’s social environment, which provides opportunities to observe and learn about the athlete. In doing so, the practitioner had a better monitoring and treatment continuum of the athletes (Henriksen et al., 2014).

#### 3.6.4 Professionals’ qualities

Several professional qualities are highlighted, such as having humility, hope, optimism, perseverance and resilience, to help support athletes (Huynh et al., 2020; Laborde et al., 2017). Having humility is to display a secure accepting identity, a self-view that is free from distortion, and an openness to new information (Huynh et al., 2020). In this line, according to Huynh et al. (2020), the humbler a coach is, the greater their influence is on their athletes. Indeed, this influence was found to be mediated by both affect-based trust and a positive team climate, which provides an environment for athlete development and success. However, coach influence was not significantly related to athletes’ cognitive trust (Huynh et al., 2020). Regarding coaching styles, using autocratic methods (e.g., harsh feedback) was associated with both disappointment and embarrassment, whereas democratic methods were associated with much higher athletes’ satisfaction (Jiménez et al., 2019).

### 3.7 Referral

Three (13.6%) studies suggested that referral was an important step toward helping athletes that are manifesting psychological distress. Indeed, when issues surpass the coaches’ scope of practice, it is advised to refer athletes to a sport psychologist or mental health professional (Lopes Dos Santos et al., 2020). In the same line, the consensus statement by Neal et al. (2015) suggested referring athletes to a mental health professional whenever needed to ensure that their apprehensions will be addressed. In cases of emergencies, like suicidal ideations or self-harm, a coach should follow standard crisis intervention procedures, while coordinating with school counselors and mental health professionals for athletes’ safety (Neal et al., 2013). In the consensus of 2013, it was advised to have a routine referral as well as a plan and protocol for emergent mental health referral, which could help student-athletes make the initial step to seek support (Neal et al., 2013).

## 4 Discussion

The objective of this scoping review was to identify how ATs can improve the psychological support provided to competitive athletes. Using the stepwise process outlined by Mak and Thomas (2022), and in accordance with the PRISMA-ScR guidelines (Moher et al., 2009), the scoping review identified 22 relevant articles. Results were divided and presented into four themes: training, prevention, intervention strategies and referral.

### 4.1 Key findings of the study

**Figure 2:**
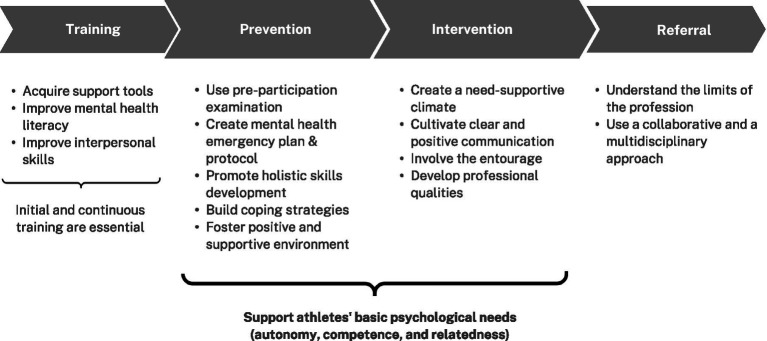
Continuum of psychological support of the competitive athletes by ATs. ATs = Athletic trainers.

Analysis of the results enabled us to map out, on a continuum, the key strategies for supporting competitive athletes experiencing psychological distress (see Figure 2). The key strategies are complementary and intertwined, so that each has a positive impact on the other.

Results suggest that training would allow ATs to acquire support tools, improve their mental health knowledge, recognize situations related to psychological problems, and develop interpersonal skills (Sebbens et al., 2016; Wilczyńska et al., 2021; Bengtsson et al., 2024; Zajonz et al., 2024). As a matter of fact, in different contexts, such as health or education, mental health literacy and better psychological support skills can help improve mental health knowledge and the ability to recognize depression and anxiety symptoms, and, therefore, better support mental wellbeing (Amado-Rodríguez et al., 2022; Malins et al., 2023). In this line of thought, training, both from an initial and continuous standpoint, is an essential ingredient for developing ATs’ skills to provide psychological support to athletes.

The next theme in the continuum refers to prevention, which relates to the implementation of early care approaches, such as preparticipation examination, and mental health emergency plan and protocol (Neal et al., 2013; Neal et al., 2015; Jasser et al., 2022; Lawrence and Gunter, 2023; Brenner and Watson, 2024). Having a plan and protocols allows to recognize, manage, and refer athletes with psychological problems more quickly and appropriately to obtain better and faster support. These findings are aligned with those of Morgan et al. (2018) for the general population, where the Mental Health First Aid (MHFA) program helped improve the intention of treatment and the amount of help provided to individuals. The MHFA is a training program that improves mental health knowledge and provides the competencies needed to help people better manage mental health problems. The implementation of other protocols and training with the same goal as MHFA, as well as screening to detect mental health risk with facilitated access to treatment, were also found to be effective in the workplace and patient care (Panagioti et al., 2017; Strudwick et al., 2023). Results about the initial spectrum of psychological care for athletes also indicated that supporting athletes in developing holistic skills and building coping strategies could help them manage difficulties related to both athletic and personal life (Tamminen and Holt, 2012; Tamminen et al., 2012; Henriksen et al., 2014; Lopes Dos Santos et al., 2020; Brenner and Watson, 2024). Similarly, Shafran et al. (2017) put forward that holistic care approaches are characterized by the care of the person, more than just the symptoms, to prevent psychological distress and negative physical health outcomes among athletes. This was also the case in high-risk professions, where coping strategies like resilience-based interventions, had a positive influence on resilience (Joyce et al., 2018).

Fostering a positive and supportive environment can also prevent athletes’ psychological distress (Tamminen and Holt, 2012; Tamminen et al., 2012; Lopes Dos Santos et al., 2020; Brenner and Watson, 2024), as well as provide guidance for intervention strategies (Henriksen et al., 2014; Jiménez et al., 2019; Lopes Dos Santos et al., 2020; Gwyther et al., 2024). One of the common points is the integration of SDT principles (Ryan and Deci, 2017), which has proven their worth in several contexts, such as sport, education and health (Ahmadi et al., 2023; Girard et al., 2023; Teixeira et al., 2020; Duda et al., 2017). In this line of thoughts, creating a need-supportive climate, which satisfy athletes’ basic psychological needs (autonomy, competence, and relatedness), is recommended for ATs (Kipp, 2014; Langan et al., 2015; Curran et al., 2016; Campbell et al., 2022; Bengtsson et al., 2024). To do so, many motivational strategies can be retrieved from scientific evidence (Duda et al., 2017; Teixeira et al., 2020; Ahmadi et al., 2023; Girard et al., 2023). For instance, to satisfy the need for autonomy, ATs could provide choices and use noncontrolling and informational language. To satisfy the need for competence, they need to provide structure and set optimal challenges. As for the need for relatedness, it is advised to consider athletes’ perspectives and use empathic listening. To support and train ATs in this matter, there is available training materials developed for other professions, such as kinesiologists,^1^ which have the potential to assist them in gaining or reinforcing their competencies on the matter. As such, the suggested motivational strategies can then be easily applied by ATs, which fits into the beginning of the continuum in terms of training.

Favoring communication (e.g., open-door policy, safe space) and involving the entourage (e.g., peers, families, coaches) during the acute management of psychological distress were identified as promising intervention strategies to support elite athletes (Henriksen et al., 2014; Braun and Tamminen, 2019; Lopes Dos Santos et al., 2020; Brenner and Watson, 2024; Gwyther et al., 2024). In fact, these kinds of intervention strategies are also recommended with psychiatric patients (Smit et al., 2023). For instance, peer-support had a positive effect on personal and clinical recovery of mental illness (Smit et al., 2023). In this line of reasoning, to increase parents’ involvement, it is advised to inform them about the purpose and the focus of the intervention.

Furthermore, according to our results, having and developing professional qualities that ATs could apply in the acute management of psychological problems in athletes is also recommended. For instance, humility, hope, optimism, perseverance and resilience are a few of the qualities that ATs could rely on to offer psychological support (Neal et al., 2013; Henriksen et al., 2014; Neal et al., 2015; Braun and Tamminen, 2019; Jiménez et al., 2019; Huynh et al., 2020). As demonstrated in our study, a practitioner who demonstrates professional qualities has a lower propensity for stigma with regards to mental health, which helps improve the attitude and incitement toward athletes (Solmi et al., 2020; Steiger et al., 2022).

Finally, referencing to other competent health care professionals allows ATs to understand the limitations of the profession and guide athletes toward appropriate care, while promoting multidisciplinary intervention approaches (Neal et al., 2013; Neal et al., 2015; Lopes Dos Santos et al., 2020). In a study conducted by Villarreal and Castro-Villarreal (2016), it was noted that multidisciplinary collaboration improved student mental health and academic outcomes. This helped to integrate treatment plans and allowed for assessment across multiple settings. Additionally, it allowed students to access services that were not available in schools and assisted in avoiding duplication of services (Villarreal and Castro-Villarreal, 2016). It is noteworthy to remember that ATs are not intended to replace mental health professionals. Nevertheless, it is imperative that ATs understand when, how and where to refer athletes for adequate psychological support, fostering the added benefits of multidisciplinary approaches.

### 4.2 Future direction

By exploring psychological management and intervention strategies we were able to target interventions for ATs to promote mental wellbeing, psychological support among elite sports athletes and mental health promotion in professional sports environments. Now, the next step could be to transform those ideas to make them practicable and actionable so that ATs can prioritize mental health alongside physical wellbeing. Further research could study the applicability and feasibility of the strategies recommended for coaches and parents for ATs, as well as their scalability in diverse sports environments. Moreover, it is imperative to integrate these approaches in initial and on-going education, so that ATs can be well-equipped and ready to deal with mental distress situations. To do so, institutions should prioritize the integration of psychological education and support into their development frameworks for ATs (Anderson et al., 2023). Through research, it would also be interesting to find concrete actions and tools for the implementation of strategies both in practice and in the training of ATs directly. It would also be relevant to create standardized documentation tools tailored for ATs, ensuring consistent application and monitoring of support strategies.

Additionally, within a framework more specific to ATs interventions, the role of psychological training in injury prevention deserves further investigation. In this line of thought, it could be relevant to look at the different approaches in helping relationships used directly by professionals managing mental health issues. Moreover, it could even pave the way to other issues such as intellectual disability in para-athletes (Domingues et al., 2024). Finally, it should be considered to widen the scope to research work interested in exceptional events, like COVID-19 (Cheng et al., 2024), that do not represent daily reality but are part of the reality of athletes. This body of literature has the potential to provide valuable information on rare situations that can exacerbate symptoms related to psychological distress.

### 4.3 Limitations and conclusion

This study has potential limitations. First, the lack of previous research studies on the topic may have limited the pool of information available on the subject. Therefore, the outcomes are partly based on studies with a lower level of scientific evidence such as clinical report, conceptual and practical application articles, dissertation, narrative review, policy recommendations’ articles and policy and guidance document based on literature review, expert opinions, and existing policies related to mental health in college sports. In addition, seven articles initially identified through the search strategy could not be consulted. A significant limitation of our study was the fact that many included studies displayed self-reported data without objective verification, leading to unclear levels of evidence for those articles. Furthermore, including studies focusing on different stakeholders, such as sports professionals (e.g., coaches, sport psychologists) and parents, limits reproducibility within the scope of the AT’s profession. Finally, as per Mak and Thomas (2022), a sixth and final step, although optional, was suggested for scoping reviews. This optional step consists of consulting stakeholders from the field of interest and was not included in this work. In fact, we suggest that this review provides sufficient data with regards to strategies to assist ATs faced with psychological distress of athletes to nourish future studies that could incorporate discussions with stakeholders. As such future avenues in knowledge translations on this topic should be encouraged to enhance professional development of ATs.

In conclusion, this study goes one step further in deepening the knowledge of ATs, which allows ATs to have a more global understanding and approach with their athletes. In this way, ATs can better support athletes suffering from psychological distress. This starts with simple and readily accessible strategies such as being available for training, being prepared for events, using strategic support interventions and knowing when to transfer psychological health care.

## Data Availability

The original contributions presented in the study are included in the article/supplementary material, further inquiries can be directed to the corresponding author.

## Footnotes

^1^see www.uqtr.ca/apprendre-a-motiver
